# Cellular Uptake and Clearance of Oxidatively-modified Apolipoprotein E3 by Cerebral Cortex Endothelial Cells

**DOI:** 10.3390/ijms20184582

**Published:** 2019-09-17

**Authors:** Siobanth Cruz, Vasanthy Narayanaswami

**Affiliations:** Department of Chemistry and Biochemistry 1250 Bellflower Blvd., California State University Long Beach, Long Beach, CA 90840, USA; scruz188@gmail.com

**Keywords:** apolipoprotein E3, lipoproteins, acrolein, endothelial cells, SRB1, LOX1

## Abstract

Apolipoprotein E3 (apoE3) plays a critical role in the metabolism of lipoproteins and lowers plasma lipid levels by serving as a ligand for the low-density lipoprotein receptor (LDLr) family of proteins and by promoting macrophage cholesterol efflux. The current study examines the effect of acrolein (an endogenously generated metabolite and an environmental pollutant) modification on the structure and function of apoE3. Acrolein modification was confirmed in Western blots by reactivity with acrolein–lysine-specific antibody and by the presence of oligomeric species due to cross-linking. LC-MS/MS analysis revealed modification of 10 out of 12 lysines in apoE3, with *N^ε^*-(3-methylpyridinium)-lysine being the predominant form of modification, and Lys75 being a ‘hot spot’ in terms of susceptibility to oxidation. Circular dichroism spectroscopy showed no major change in overall secondary structure compared to unmodified apoE3. Reconstituted high density lipoprotein (HDL) bearing acrolein modified apoE3 showed loss of binding to soluble LDLr; however, incubation with mouse endothelioma bEnd.3 cells showed that it was internalized. Incubation with excess LDL did not abolish cellular uptake of acrolein modified apoE3, suggesting alternative mechanism(s) not involving LDLr. Incubation with anti-CD36 antibody did not show a decrease in internalization while incubation with anti- lectin-like oxidized LDL receptor 1 (LOX1) showed partial internalization. However, incubation with anti-scavenger receptor class B type I (SRB1) antibody abolished internalization of acrolein modified apoE3. Taken together, our studies suggest that acrolein modification of apoE3 at lysine residues leads to increase in net negative charge, and as a consequence, results in clearance by LOX1 and SRB1 on endothelial cells. Overall, oxidative modification of apoE3 likely impairs its role in regulating plasma cholesterol homeostasis, eventually leading to lipid disorders.

## 1. Introduction

Aging and several disease states such as neurodegenerative diseases, cardio- and cerebrovascular diseases, and inflammation are characterized by heightened cellular and plasma oxidative stress accompanied by increased levels of reactive oxygen and nitrogen species (reviewed in [1]. Plasma lipoproteins are particularly prone to oxidative damage due to their high lipid content, with oxidized low-density lipoproteins (ox-LDL) being an outstanding example for their role in the formation of atherosclerotic plaques that lead to heart disease and stroke [2]. Formation of plaques is the consequence of accumulation of calcium, lipids such as cholesterol, cellular waste, and other biomolecules in the inner lining of the artery in the sub-endothelial space [2]. A monolayer of endothelial cells line the vasculature forming a junction between the blood and underlying tissue; it constitutes the first line of encounter of ox-LDL at the cellular level, with endothelial dysfunction being an early event in atherogenesis [3].

In the brain, plaque formation in the cerebral microvasculature is a major underlying cause of vessel narrowing that leads to restricted blood flow and ischemic stroke [4]. Whereas the role of oxidative stress and ox-LDL in the etiology of atherosclerosis [5] and ischemic stroke is well-established [4,6], that of oxidized high density lipoprotein (ox-HDL), and, associated proteins and lipids have gained attention only in recent years (reviewed in [7]). This is particularly relevant as lipoprotein researchers have made a paradigm shift in focus from identifying approaches to increasing HDL cholesterol levels (for improved cardiovascular outcomes) to improving the quality and functionality of HDL [8]. HDL is associated with multiple functions including ability to promote cholesterol efflux, antioxidative, anti-inflammatory and anti-thrombotic properties [8,9,10,11]. Several of these functions have been attributed to the major protein component of HDL, apolipoprotein AI (apoAI) through studies with native and reconstituted HDL (rHDL) [8]. However, the contribution of other important apolipoprotein components such as apolipoprotein E3 (apoE3) in HDL sub-fractions to loss of HDL function is less understood. The goal of the current study is to understand the contribution of apoE3 towards generating ox-HDL. ApoE3 is well known for its critical role in maintaining plasma cholesterol and triglyceride homeostasis and is considered an anti-atherogenic protein [12,13,14]. It also plays a crucial role cholesterol efflux and reverse cholesterol transport [15], and bears anti-oxidant and anti-inflammatory properties [16]. In addition, it contributes towards maintaining the integrity of the blood–brain barrier (BBB) [17], and endothelial function at the neurovascular junction [18]. These studies report that apoE-null mice display increased endothelial cell permeability, with aging exacerbating the process.

A key functional feature of apoE3 is its ability to serve as a ligand for the LDL receptor (LDLr) family of proteins [13] that leads to clearance and internalization of the lipoprotein particles, thereby lowering plasma cholesterol levels [13,19,20]. It is an exchangeable apolipoprotein that has the ability to exist in lipid-free and lipoprotein-associated states [21]. In the latter state, it is primarily located on plasma lipoproteins such as chylomicron remnants, very low density lipoprotein and HDL; among the latter two, apoE3 elicits a preference for HDL over VLDL [22]. In the lipid-free state, it is comprised of two independently folded domains: A 22 kDa N-terminal (NT) domain (residues 1–191) housing the LDLr-binding sites [19] and a 10 kDa C-terminal (CT) domain (residues 210–299) bearing high-affinity lipid binding sites [19]. The two domains are linked by a protease-sensitive loop. In the lipid-free state, apoE3 undergoes protein-protein interaction via its CT domain giving rise to dimers, tetramers and higher state oligomers. High resolution structural analysis of a monomeric variant of apoE3 revealed a four-helix bundle in the NT domain [19], comprised of helices H1, H2, H3, and H4. A constellation of Lys and Arg residues that is localized on helix H4 and its vicinity constitutes the LDLr binding site [19].

The objective of the current study is to examine the fate of oxidatively modified apoE3, specifically acrolein-modified apoE3, in cerebral cortex endothelial cells. Preliminary evidence from our lab suggested presence of acrolein-modified apoE in human plasma. Acrolein is a highly reactive α,β-unsaturated aldehyde that is generated endogenously as a product of lipid peroxidation, polyamine and drug metabolism and is also found in the environment as a pollutant [23]. We determined that direct exposure of purified recombinant apoE3 to acrolein causes impairment in LDLr binding ability, and attributed the loss to modifications of Lys residues that lead to localized changes in the vicinity of LDLr binding sites. Further, we noted that acrolein-modified apoE3 was internalized by alternate routes by brain endothelial cells, serving as (hitherto unknown) ligands for scavenger receptors, similar to those reported for ox-LDL.

## 2. Results

### 2.1. Acrolein Modification of apoE3

**Figure 1:**
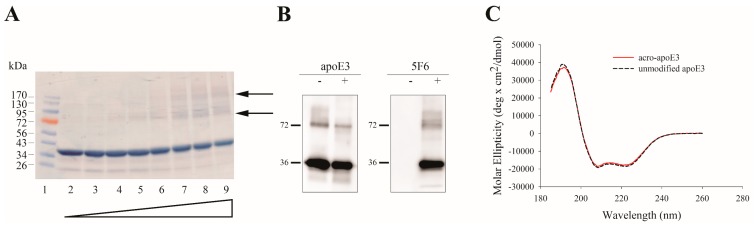
Characterization of acrolein modification of apolipoprotein E3 (apoE3). (**A**) SDS-PAGE of acrolein modified apoE3. About 10 µg of apoE3 was treated with PBS or increasing amounts of acrolein, electrophoresed on a 4–20% acrylamide gradient gel and stained with Amido Black. The lane assignments for the various acrolein:apoE3 molar ratios are as follows: lane (1) standard, lane (2) unmodified apoE3, lane (3) 1:1, lane (4) 10:1, lane (5) 20:1, lane (6) 40:1, lane (7) 60:1, lane (8) 80:1, lane (9) 100:1. Arrows draw attention to 72 and 130 kDa bands. (**B**) Western blot of unmodified and acro-apoE3. About 0.5 µg unmodified or acro-apoE3 was electrophoresed and subjected to Western blot using anti-apoE-HRP (left panel) and 5F6 (right panel) antibody. A molar ratio of 10:1 acrolein:apoE3 was used for Western blot. (**C**) Far-UV CD spectra of unmodified apoE3 and acro-apoE3. Far-UV CD spectra of about 0.2 mg/mL protein were recorded in 10 mM ammonium bicarbonate buffer at pH 7.4, under reduced conditions, in the presence of Tris-(2-carboxyethyl) phosphine (TCEP).

Purified apoE3 was treated with increasing amounts of acrolein in 20 mM sodium phosphate buffer, pH 7.4 containing 150 mM NaCl (phosphate buffered saline, PBS) or PBS alone, followed by dialysis to remove unbound acrolein. SDS-PAGE analysis under reducing conditions (Figure 1A) revealed a major monomeric band at ~36 kDa, in addition to minor bands at 72 and 130 kDa with increasing acrolein, the latter two corresponding to dimeric and oligomeric species. This was accompanied by a decrease in intensity of the monomeric band. In subsequent studies we used 10:1 ratio of acrolein:apoE3 (~1 acrolein/Lys), the rationale being that there are 12 lysine residues in apoE3 and that acrolein is most reactive with Lys. For cell-based studies, 1–5 µg /mL unmodified or acro-apoE3 (equivalent to <1 µM of apoE3) was used. These concentrations are within the range (µM) reported for in vivo values of protein-conjugated acrolein. Figure 1B shows Western blots of apoE3 modified by acrolein at 10:1 ratio and detected by apoE-HRP (left panel) or mAb5F6, anti-acrolein–lysine antibody (right panel). At this ratio, there was evidence of apoE3 modification by acrolein as revealed by a major band at ~36 kDa (right panel) in addition to a significant covalently cross-linked dimeric band. As expected, the 5F6 antibody does not detect unmodified apoE3 as shown in control experiments (Figure 1B, right panel), confirming its specificity for acrolein modified proteins. The band at ~72 kDa observed in unmodified apoE3 (Figure 1B, left panel), is likely due to the presence of SDS-resistant dimeric species. Taken together, these results confirm that acrolein modification of apoE3 generates epitopes that are recognized by 5F6. The monomeric band is indicative of acrolein adduct formation without intermolecular cross-linking. In addition, the presence of higher molecular weight bands in the Western blot is indicative of acrolein-mediated intermolecular cross-linking.

Far-UV circular dichroism (CD) spectra of acro-apoE3 showed typical features of a highly helical protein characterized by troughs at 208 and 220 nm (Figure 1C), similar to that noted for unmodified protein. The α-helical content of acro-apoE3 was calculated to be 53 ± 2% while that for unmodified apoE3 was 55 ± 3%.

### 2.2. Mass Spectral Analysis

**Figure 2:**
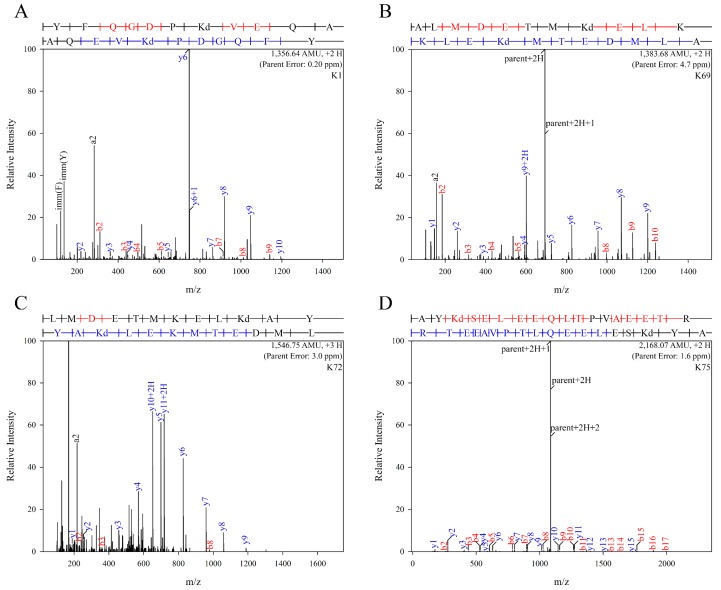

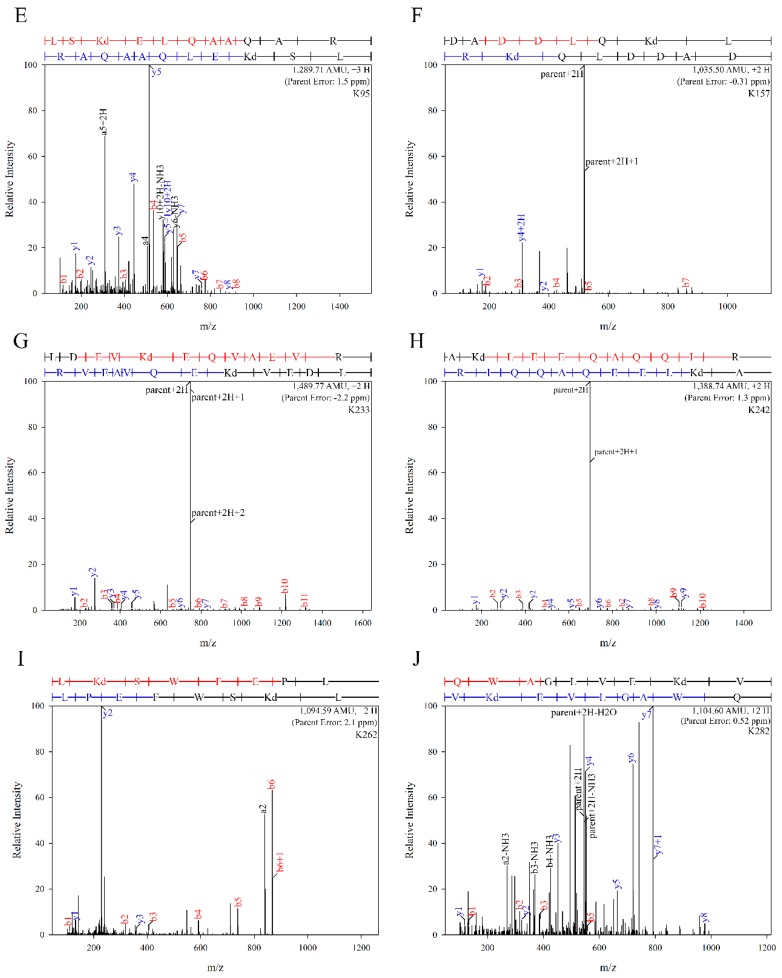
LC-MS/MS profiles of major modified sites in acro-apoE3. Purified apoE3 was treated with acrolein, subjected to in-gel digestion and analyzed by nano LC-MS/MS as described under Materials and Methods and Supplemental Information. The panels show spectra of peptides where major acrolein modification of the different sites were observed: (**A**): K1; (**B**): K69; (**C**): K72; (**D**): K75; (**E**): K95; (**F**): K157; (**G**): K233; (**H**): K242; (**I**): K262; and, (**J**): K282.

To identify the nature of modification and specific residues that were modified by acrolein, unmodified and acro-apoE3 were subjected to mass spectral analysis. Protease treatment of unmodified and acro-apoE3 yielded a sequence coverage of 96% for trypsin and 97% for elastase, with a combined coverage of 99% for both proteases, Figure S1. Figure 2 shows the MS/MS spectra of peptides from acro-apoE3 with mass increases above those expected from unmodified apoE3. Table 1 shows sites and nature of modification in acro-apoE3 following trypsin and elastase digestion and LC-MS/MS analysis. Only those sites identified in acro-apoE3 but not in unmodified apoE3 (i.e., spectral count = 0 for corresponding site in unmodified apoE3) or for which the spectral score was higher in acro-apoE3 than that for unmodified apoE3 were considered. Of the 12 Lys in apoE3 (K1, K69, K72, K75, K95, K143, K146, K157, K233, K242, K262, and K282) all except K143 and K146 were modified by acrolein in one or more forms, showing mass increases of +38, +56, +76 and +94, corresponding to aldimine, propanal, N^ε^-(3-methylpyridinium)-lysine (MP-Lys), and N^ε^-(3-formyl-3,4-dehydropiperidino)-lysine (FDP-Lys) adducts, respectively. Interestingly, K75 appeared to be highly susceptible to oxidative modification by acrolein displaying all 4 types of mass increase.

### 2.3. LDLr Binding Ability of acro-apoE3/rHDL

**Figure 3:**
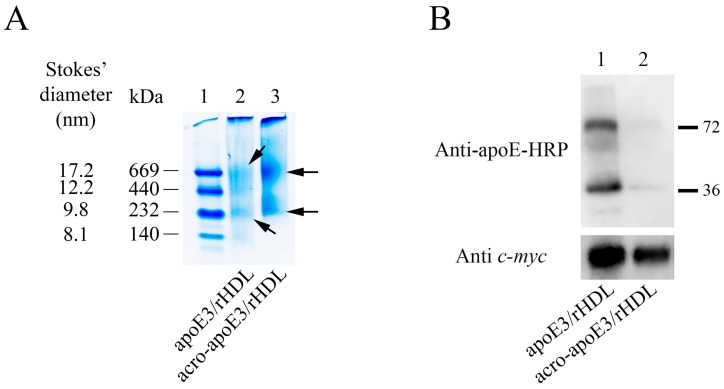
Characterization of lipid-associated unmodified and acro-apoE3. (**A**) Non-denaturing PAGE of apoE3/rHDL and acro-apoE3/rHDL. The rHDL particles (~20 µg protein) were electrophoresed on a 4–20% acrylamide gradient. The lane assignments are as follows: lane (1) high molecular mass standards, lane (2) apoE3/rHDL and lane (3) acro-apoE3/rHDL. Arrows draw attention to rHDL bands in unmodified and acro-apoE3. The Stokes’ diameter and molecular masses of the reference proteins (from top, thyroglobulin, ferritin, catalase, and albumin) are shown. (**B**) Effect of acrolein modification on sLDLr binding ability of apoE3/rHDL or acro-apoE3/rHDL. ApoE3/rHDL or acro-apoE3/rHDL (10 µg protein) was incubated with 10 µg of sLDLr at 4 °C for 1 h, followed by co-IP with anti-*c-myc* agarose. ApoE3 bound to sLDLr was detected by Western blot using HRP-conjugated polyclonal apoE antibody (Top). sLDLr was detected by anti-*c-myc* antibody for comparison (Bottom). The lane assignments are as follows: Lane (1) apoE3/rHDL; lane (2) acro-apoE3/rHDL.

Since mass spectral data indicate that Lys residues (K143 and K146) located directly in the LDLr binding region were not modified, a spot check was performed to assess the LDLr binding ability of acro-apoE3 in solution using co-IP. As the LDLr binding ability of apoE3 is elicited only in the lipid-bound state of the protein, unmodified or acro-apoE3 was reconstituted with POPC to generate rHDL as described previously [24]. Acrolein modification did not affect the ability of apoE3 to form lipid complexes with POPC. Non-denaturing PAGE analysis revealed major bands of ~669 kDa and ~232 kDa for particles containing either unmodified or acro-apoE3 (Figure 3A). Subsequently, apoE3/rHDL or acro-apoE3/rHDL was incubated with soluble LDLr (sLDLr) bearing ligand binding domains LA3-LA6 with a *c-myc* epitope, the receptor-bound complexes captured by anti-c-*myc*-agarose and the bound complex detected by anti-apoE-HRP or anti-c-*m*yc antibody (Figure 3B). The data show that under conditions where unmodified apoE3 binds robustly to sLDLr (Figure 3B, Top, lane 1), acrolein modification impairs its ability to bind to sLDLr. The band ~72 kDa corresponds to the SDS-resistant dimeric species. The bottom panel shows Western blot of the bound complex, which was probed with anti-c-*myc* antibody, demonstrating the presence of sLDLr in both lanes 1 and 2.

### 2.4. Cellular Uptake of acro-apoE3/rHDL

**Figure 4:**
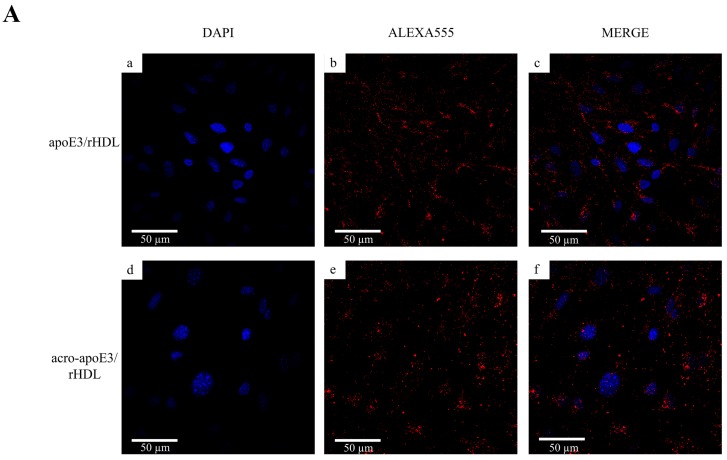

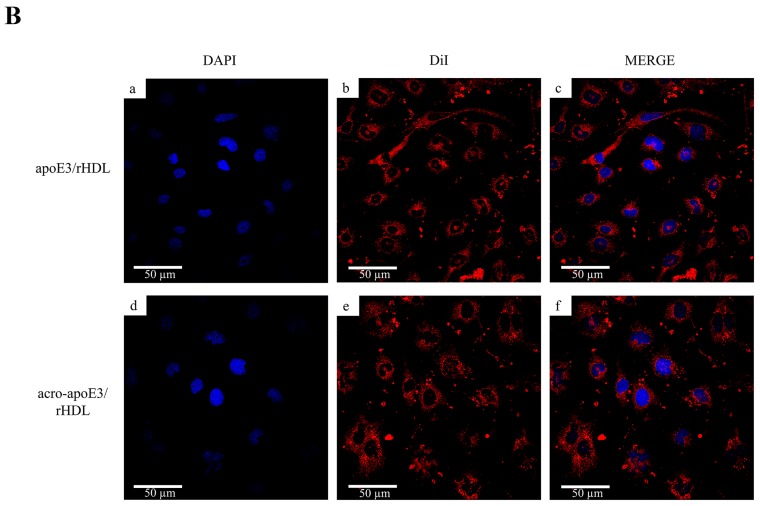
Uptake of apoE3/rHDL and acro-apoE3/rHDL by bEnd.3 cells. (**A**) Uptake followed by immunofluorescence. Uptake of rHDL was visualized by immunofluorescence following exposure to 3 µg/mL apoE3/rHDL (a–c) or acro-apoE3/rHDL (d–f) for 2 h at 37 °C. (**B**) Uptake followed by direct fluorescence of DiI. Uptake experiments were carried out as above in the presence of DiI-labeled apoE3/rHDL (a–c) or acro-apoE3/rHDL (3 µg/mL) (d–f). The panels show fluorescence of DAPI (a,d), DiI (b,e), and Merge (c,f).

In the next step, cellular uptake of apoE3/rHDL and acro-apoE3/rHDL was investigated in bEnd.3 cells. The uptake was followed by immunofluorescence using anti-apoE antibody, 3H1 and Alexa-555 labeled secondary antibody (Figure 4A). Perinuclear punctate vesicles were observed for both apoE3/rHDL and acro-aproE3/rHDL indicative of cellular uptake by receptor-mediated endocytosis.

In parallel, the uptake of the lipid component was visualized by direct fluorescence using DiI-labeled lipoprotein particles (Figure 4B). A similar punctate distribution of endocytic vesicles was noted for both DiI-labeled apoE3/rHDL and acro-apoE3/rHDL. While the co-IP data indicated that acro-apoE3/rHDL was unable to bind to sLDLr, immunofluorescence data showed that both the protein and lipid components of acro-apoE3/rHDL were internalized by the cells. This suggested that acro-apoE3/rHDL likely binds to receptor(s) other than LDLr and that the cells adopt an alternative route of particle uptake.

**Figure 5:**
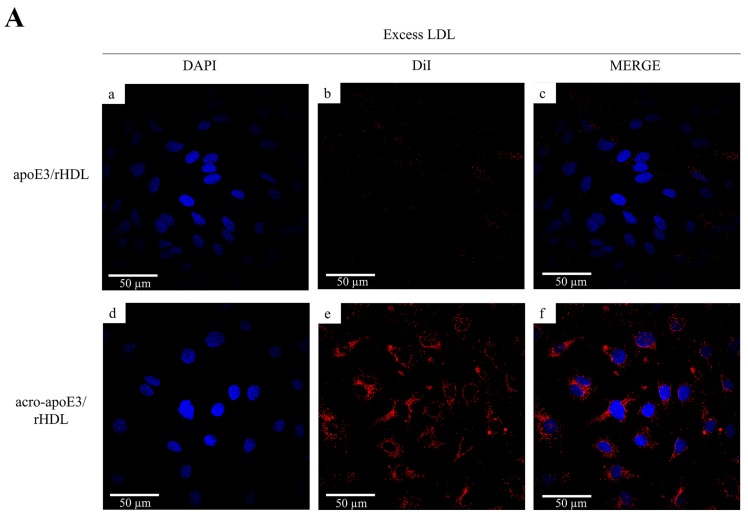

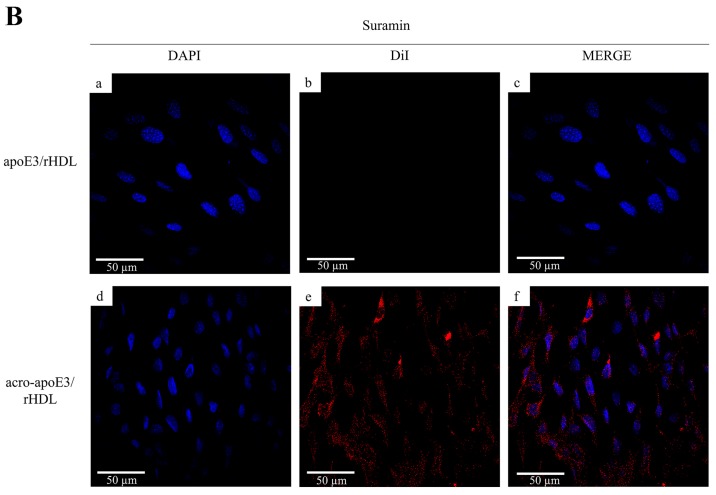
Uptake of apoE3/rHDL and acro-apoE3/rHDL by bEnd.3 cells. Uptake was followed by direct fluorescence in the presence of excess LDL (**A**) or suramin (**B**). Uptake experiments were carried out in the presence of 100× excess LDL or 2mM suramin and apoE3/rHDL (a–c) or acro-apoE3/rHDL (d–f) (apoE3 concentration: 3 µg/mL). The panels show fluorescence of DAPI (a,d), DiI (b,e), and Merge (c,f).

To investigate this aspect further we designed a series of experiments to definitively exclude the role of LDLr and to explore the possibility of involvement of other known receptors. Initial studies assessed the effect of excess LDL to competitively inhibit the uptake via LDLr, based on the ability of apoB100, the major non-exchangeable apolipoprotein on LDL, to serve as a ligand for the LDLr. In control reactions, the addition of 100x excess LDL over apoE3 inhibited cellular uptake of apoE3/rHDL, but not that of acro-apoE3/rHDL (Figure 5A). Similarly, the uptake of apoE3/rHDL, but not acro-apoE3/rHDL, was inhibited by suramin, an inhibitor of LDLr (Figure 5B). This observation confirms that oxidatively-modified apoE3 does not bind and is not internalized by the LDLr, but is likely taken up by alternative routes.

### 2.5. Internalization of Oxidatively Modified apoE3 by an Alternative Pathway

**Figure 6:**
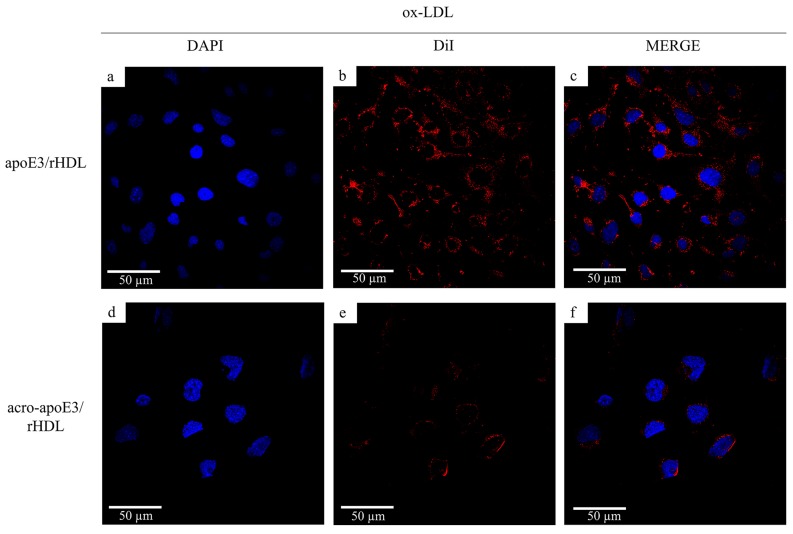
Uptake of apoE3/rHDL and acro-apoE3/rHDL in the presence of ox-LDL. Uptake experiments were carried out as described earlier with 3 µg/mL apoE3/rHDL (**a**–**c**) or acro-apoE3/rHDL (**d**–**f**) in the presence of 100× excess ox-LDL (300 µg/mL). The panels show fluorescence of DAPI (a,d), DiI (b,e), and Merge (c,f).

It is well established that endothelial cells internalize modified LDL by scavenger receptors, which display broad ligand specificity (including polyanionic species such as nucleic acids, polysaccharides, and phospholipids) [25,26,27,28,29]. To investigate the possibility that HDL containing oxidatively modified apoE3 can also be internalized by these receptors, the effect of competition by ox-LDL, a physiological ligand for scavenger receptors, was examined. When cells were treated with 100× excess ox-LDL, the uptake of DiI-labeled apoE3/rHDL was not affected while that of acro-apoE3/rHDL was significantly reduced (Figure 6). The reduction in intracellular fluorescence suggests the involvement of scavenger receptors such as lectin-like oxidized LDL receptor 1 (LOX1), CD36 and/or SRB1. All three have been known to bind oxidized HDL or any modified species in circulation: CD36 has been shown to internalize minimally oxidized LDL [27], copper oxidized HDL but not native HDL [30]. SRB1 has been reported to bind to acrolein modified HDL [31], while LOX1 is considered the receptor of ox-LDL [32]. Fucoidan, a negatively charged polysaccharide that serves as a conventional ligand for class A scavenger receptors, showed no significant decrease in the uptake of acro-apoE3/rHDL (Figure S2) suggesting that class A receptor was likely not involved; when treated with carrageenan, uptake of acro-apoE3/rHDL decreased by ~80% (Figure S2).

**Figure 7:**
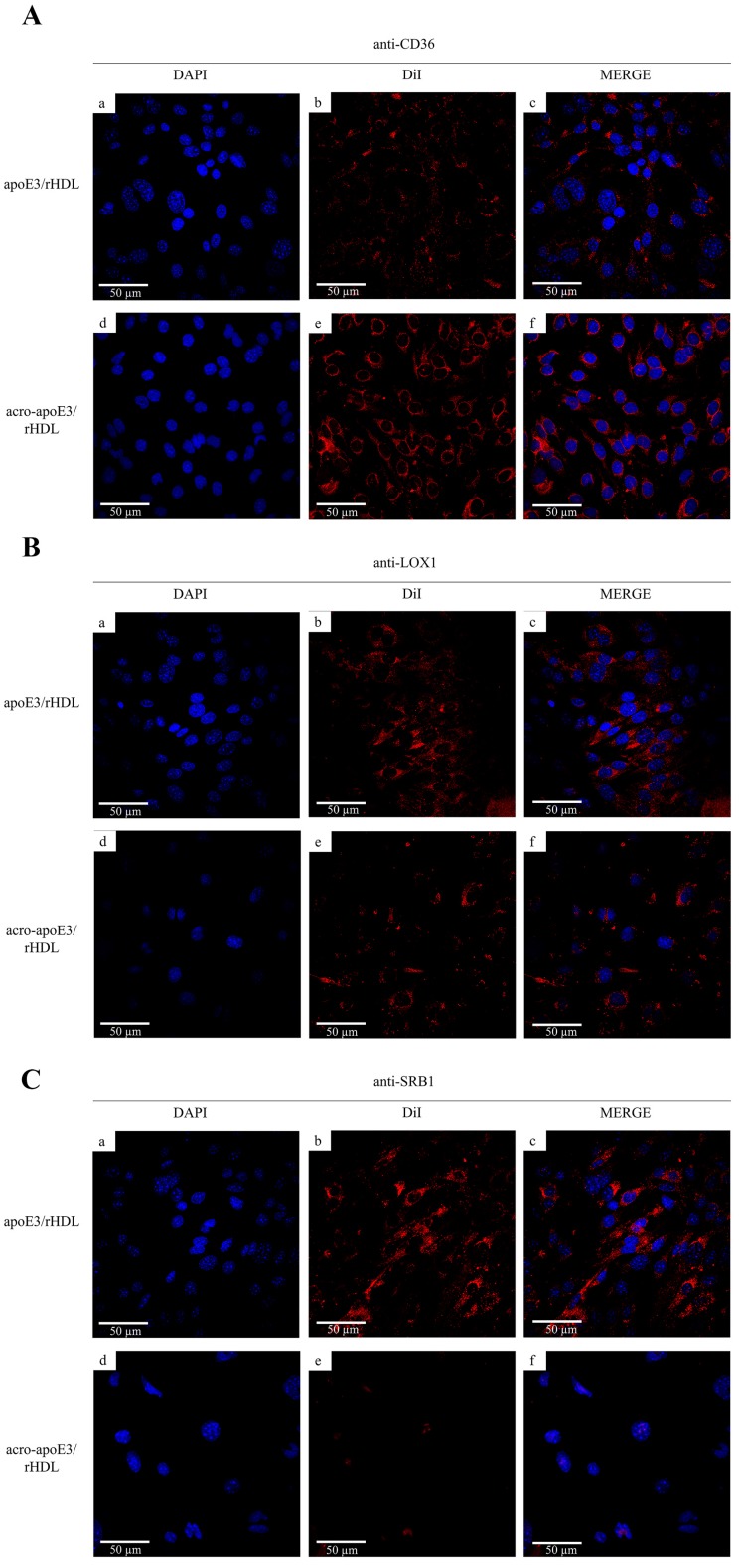
Uptake of apoE3/rHDL and acro-apoE3/rHDL in the presence of anti-CD36, anti-LOX1 or anti-SRB1 antibody. Uptake experiments were carried out for 2 h at 37 °C with 3 µg/mL apoE3/rHDL (**a**–**c**) or acro-apoE3/rHDL (**d**–**f**) in the presence of 100 µg anti-CD36 (**A**), anti-LOX1 (**B**) or anti-SRB1 (**C**) antibody. The panels show fluorescence of DAPI (a,d), DiI (b,e), and Merge (c,f).

Lastly, the cells were pre-treated with anti-CD36, anti-LOX1 or anti-SRB1 antibodies (Figure 7), prior to exposure to DiI-labeled apoE3/rHDL or acro-apoE3/rHDL. Interestingly anti-CD36 antibody reduced the uptake of apoE3/rHDL but had no effect on the uptake of acro-apoE3/rHDL (Figure 7A). Anti-LOX1 antibody reduced the uptake of acro-apoE3/rHDL but not that of apoE3/rHDL (Figure 7B). The presence of anti-SRB1 antibody significantly reduced the uptake of acro-apoE3/rHDL while partially affecting the uptake of apoE3/rHDL (Figure 7C). Together, the data suggest the involvement of LOX1 and SRB1 in the internalization of acro-apoE3 in bEnd.3 cells.

## 3. Discussion

The cerebral microvasculature is susceptible to oxidative stress, which contributes significantly to ischemic injury and stroke [4]. Whereas the role of ox-LDL in the etiology of atherosclerosis and ischemic stroke [6] has been recognized for decades, that of ox-HDL has only gained attention over the last decade, and very little is known about its role at the neurovascular junction. The objective of the study is to investigate the fate of oxidatively modified apoE3 when exposed to endothelial cells lining the cerebral vasculature; apoE3 is one of the critical anti-atherogenic protein components on VLDL, VLDL remnants and a sub-fraction of HDL. It is not known if oxidized apoE3 is cleared by similar mechanisms as those employed for ox-LDL. Using acrolein as a model oxidative stressor, our studies demonstrate that: (i) acrolein forms aldimine, propanal, MP-Lys, and FDP-Lys adducts on 10 Lys residues in apoE3, (ii) acrolein modification impairs ability of apoE3-bearing rHDL to bind soluble LDLr and be internalized by LDLr on bEnd.3 cells, and, (iii) acrolein-modified apoE3 is internalized by LOX1 and SRB1 on bEnd.3 cells.

Oxidative stress impairs the integrity of the blood–brain barrier [33], which is a tightly regulated interface at the neurovascular junction between the brain and the vasculature. It is comprised of a layer of endothelial cells that restrict the passage of potentially neurotoxic plasma components, blood cells, environmental and metabolic toxins, and pathogens into the brain [34]. A crucial aspect in preventing atherogenesis and other vascular diseases is the ability to effectively clear ox-LDL by macrophages, vascular endothelial cells, and activated smooth muscle cells. It is a protective mechanism to prevent build-up of cholesterol containing atherosclerotic plaques. This is facilitated by scavenger receptors, the presence of which is one of signature features of endothelial cells.

### 3.1. Acrolein Forms Aldimine, Propanal, MP-Lys, and FDP-Lys Adducts in apoE3

Acrolein is the strongest electrophile among all α,β-unsaturated aldehydes and can form covalent adducts with Lys, Cys, and His. Significantly high levels (1-100 µM range) of acrolein and/or protein-conjugated acrolein have been reported in patients with Alzheimer’s disease, mild cognitive impairment, cancer subjects receiving oxazaphosphorine drugs, and those with renal failure [23,35,36,37,38]. Acrolein adduct formation has been demonstrated in lipid-free apoAI, HDL-bound apoAI [39,40], and apoB-100 in LDL [41]. Protein-bound acrolein arising from endogenous lipid peroxidation and polyamine metabolism has been detected in fatty streak lesions in arterial vessels [42], raising the possibility that they may be used as potential markers of oxidative damage and atherosclerosis [43].

**Figure 8:**
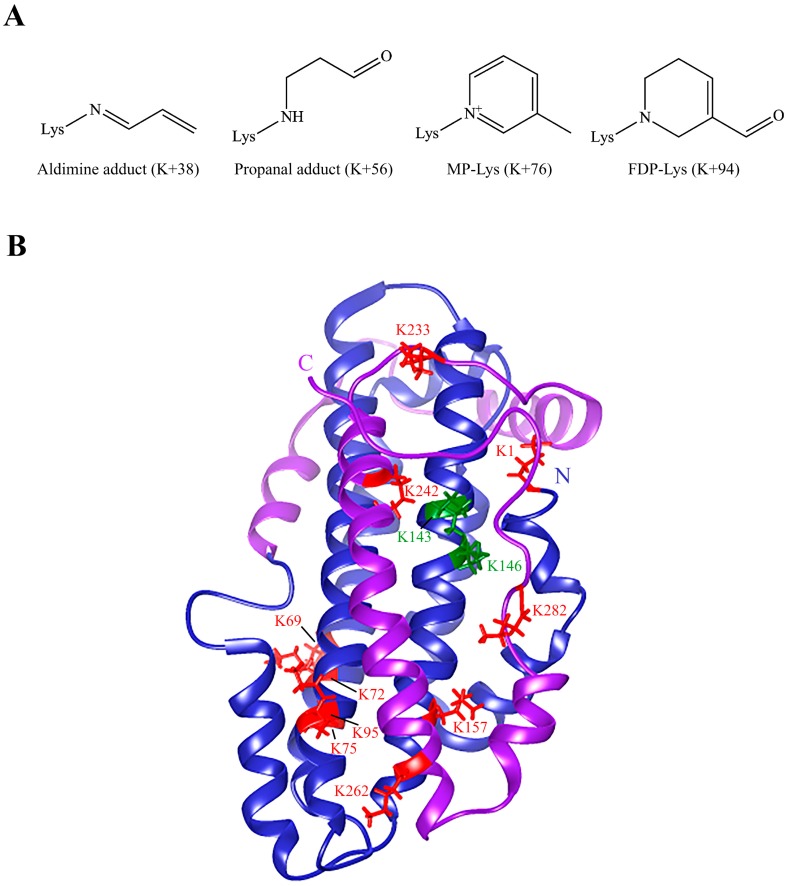
Acrolein modification of apoE3. Panel (**A**) Structures of acrolein adducts identified in acro-apoE3. The numbers in parentheses indicate the observed increase in mass units based on mass spectral analysis. (**B**) Structure of apoE3 showing acrolein-modified Lys. The overall organization of a monomeric form of apoE3 (PDB # 2L7B) is shown. The image was generated using UCSF Chimera visualization system (http://www.rbvi.ucsf.edu/chimera). All Lys residues are shown; red depicts all acrolein-modified Lys, while green depicts Lys residues that were most likely not modified. Blue ribbon: NT domain; purple ribbon: CT domain.

In initial studies, we noted that exposure to acrolein generated epitopes on apoE3 that are recognized by acrolein–lysine-antibody, developed by Uchida and colleagues [42], leading to adduct formation as monomers and cross-linked dimers. This suggested that apoE3′s ability to interact with LDLr may be impaired directly or indirectly. The mass increase, and the corresponding names and structures of the adducts that were derived from a survey of the post translational modifications data base are shown in Figure 8A.

The mass increase of +56 was attributed to propanal adduct formation with Lys when acrolein undergoes nucleophilic addition at the double bond leading to a Michael addition-type adduct [41]. The mass increase of +38 corresponds to Schiff base formation between acrolein’s aldehyde group and the ε-amino group of Lys; this, in turn, reacts with a second acrolein via a Michael addition cyclizing to MP-Lys (+76) via an imine intermediate [44]. Lastly, the +94 mass increase is the consequence of reaction of Lys with 2 acrolein molecules by Michael addition followed by condensation and dehydration reactions [41,42,45,46]. An interesting observation was that K143 and K146, which lie directly in the LDLr binding region on helix H4 (130–150), were neither modified nor cleaved, regardless of the protease used (trypsin, elastase). The lack of reactivity may be attributed to the unusually low pK_a_ of 9.5 and 9.2 for K143 and K146, respectively, which contribute to the strong positive electrostatic potential in their microenvironment [47,48] or to shielding by the CT domain. Intriguingly, when rat apoE was treated with acrolein [49], K135 and K138 (corresponding to K143 and K146 in human apoE3) were modified as propanal adducts leading to loss of heparin- and LDLr-binding activities. Figure 8B shows all the Lys residues in apoE3 that were modified by any type of acrolein modification.

### 3.2. Acro-apoE3/rHDL Does Not Interact with sLDLr and LDLr on bEnd.3 Cells

Previous studies from our lab using isolated rat apoE demonstrated loss of sLDLr binding activity suggesting acrolein modification of Lys residues in helix H4 [49]. In subsequent studies, we demonstrated that exposure of rats to second hand tobacco smoke leads to acrolein modification of plasma apoE in vivo [49], with in vitro data suggesting that acrolein forms an aldimine adduct at K149 and K155, a propanal adduct at K135 and K138, an MP-lysine at K64, K67, and K254 and FDP-Lys at K68 [49]. Considering that 2 of these Lys residues (K135 and K138) are located directly in the LDLr binding region it was not surprising that acrolein modification of rat apoE lead to loss of sLDLr binding activity [49]. In contrast, the current mass spectral data of acrolein-modified apoE3 revealed that none of the modified Lys residues lie within the LDLr binding region and yet the sLDLr binding ability was compromised. These findings suggest that in addition to those located on helix H4, residues lying outside of H4 contribute substantially to LDLr binding in apoE3.

To test the ability of acro-apoE3 to bind to sLDLr or to LDLr on endothelial cells, lipid bound forms were generated with POPC to yield acro-apoE3/rHDL. There were no significant differences in the particle size between the apoE3/rHDL and acro-apoE3/rHDL in non-denaturing PAGE, with both preparations revealing 2 major populations. To assess internalization of acro-apoE3 by cellular LDLr, apoE3/rHDL or acro-apoE3/rHDL were exposed to bEnd.3 cells. Interestingly, we noted uptake of acro-apoE3 by bEnd.3 cells under experimental conditions typically employed for LDLr-mediated uptake. The uptake was similar to that noted for the unmodified protein, and was observed regardless of whether the protein or lipid component was monitored. In competition assays, uptake of apoE3/rHDL, but not of acro-apoE3/rHDL, was abolished in the presence of excess LDL or suramin, confirming that oxidatively modified apoE does not interact with LDLr. Together, it leads us to propose that acrolein adduct formation on Lys residues likely has an indirect effect by altering the conformation of the LDLr binding region of apoE3 (for example the curvature of the segment that interacts with the LDLr) [50] and therefore its interaction with the ligand binding domain of sLDLr. We further postulated that oxidative modification of apoE3 by acrolein likely leads to uptake via scavenger receptor pathway(s).

### 3.3. Acrolein-Modified apoE3 Is Internalized by LOX1 and SRB1 on bEnd.3 Cells

The premise for the above postulate was based on our mass spectral data that identified 10 out of 12 Lys had acrolein adducts, which likely increased the net negative charge of apoE3, thereby making acro-apoE3/rHDL eligible to serve as a ligand for scavenger receptors. It is well established that scavenger receptors recognize and internalize macromolecules that have a net negative charge, including oxidized and acetylated LDL, and participate in the removal of a wide range of biomolecules such as lipids (phosphatidylserine, fatty acids, cholesterol), modified proteins (β-amyloid fibrils), native proteins (chaperones, cytokinases) and dead cells/debris or apoptotic cells [25,51].

The observation that ox-LDL served as a competitive inhibitor and significantly decreased the uptake of acro-apoE3/rHDL, but not apoE3/rHDL, in bEnd.3 cells suggested that acro-apoE3/rHDL is likely internalized by a pathway similar to that used for ox-LDL. Since ox-LDL is internalized by CD36, we investigated its involvement by using a monoclonal antibody that specifically binds to residues 155–183. We noted that the uptake of acro-apoE3/rHDL did not decrease significantly; intriguingly, the uptake of unmodified apoE3/rHDL decreased by ~70% in the presence of anti-CD36 antibody, the reasons for which are not clear. The decrease may be attributed to the protein or lipid component being air oxidized and recognized by CD36. Since CD36 is able to recognize moderately oxidized LDL and oxidized phospholipids [52,53,54] it is possible that our preparations of apoE3/rHDL have been slightly oxidized. Since the uptake of acrolein modified apoE3 was not significant in the presence of anti-CD36 antibody, we excluded CD36 as a possible route for uptake of acro-apoE3/rHDL.

Other studies have shown that ox-LDL can be internalized by LOX1 [55], a receptor identified in bovine and human aortic endothelial cells. When anti-LOX1 antibody was used, there was an 80% decrease in the uptake of acro-apoE3/rHDL, suggesting that acro-apoE3/rHDL is a ligand for LOX1. LOX1 belongs to the class of scavenger receptors, and plays a critical role in initiation and progression of atherosclerosis by mediating dysregulation of endothelial cell function, triggering a pro-inflammatory response following ligand binding [56]. The ability of acro-apoE3 to bind LOX1 is consistent with the pattern of ligands reported for this scavenger receptor, such as modified LDL, C-reactive protein, and an electronegative lipoprotein fraction, L5, in human plasma.

Another possibility of clearance includes SRB1, which shares sequence similarities with CD36 in the extracellular loop domain, including conserved cysteines and multiple N-linked glycosylation sites [57,58]. Known as the HDL receptor, SRB1 is able to mediate selective uptake of cholesteryl esters from HDL and LDL, and to facilitate cholesterol efflux to lipoprotein acceptors. One of the major roles of SRB1 is to mediate cholesterol uptake and facilitate reverse cholesterol transport [57,58]. SRB1 appears to interact with native and oxidized lipoproteins in significantly different ways; it bears an increased number of binding sites and binds ox-LDL with a higher affinity than native HDL [26,29,30]. Further, whereas ox-LDL can be internalized in its entirety, native LDL experiences selective uptake of its cholesteryl ester core. In the case of HDL, the binding affinity is higher and the core selective uptake is lower in acrolein-modified HDL than native HDL [31]. Our current data provide further information about the role of SRB1 in its ability to bind acro-apoE3/rHDL in bEnd.3, as inferred from the abolition of its uptake by anti-SRB1 antibody. Acro-apoE3/rHDL likely engages SRB1 in a manner that leads to uptake of lipid components since a decrease in DiI fluorescence was noted. Future studies are needed to determine the exact nature of interaction between oxidized apoE3 and SRB1 or LOX1, which likely serve as a first line of defense against the potentially damaging effects of circulating oxidized lipoproteins on the walls of the cerebral microvasculature. Studies are also needed to determine the pro-inflammatory response of endothelial cells triggered by oxidatively modified apoE3 and its potential role in plaque development at the neurovascular junction.

## 4. Materials and Methods

### 4.1. Acrolein Modification of apoE3

Recombinant apoE3 bearing a hexa-His tag at the N-terminal end and a TEV protease cleavage site was generated as described previously [24]. In initial studies purified apoE3 was treated with varying concentrations of a stock solution of 15 mM acrolein (MilliporeSigma, St Louis, MO, USA) in 20 mM sodium phosphate buffer, pH 7.4 containing 150 mM NaCl (phosphate buffered saline, PBS) for 4 h at 37 °C. In subsequent studies, the acrolein:apoE3 molar ratio was maintained at 10:1. In control reactions, apoE3 was incubated with PBS with no added acrolein to account for modifications during handling process. Excess unreacted acrolein was dialyzed out against PBS for 48 h with three changes. The unmodified and acrolein modified apoE3 (acro-apoE3) were electrophoresed on a 4%–20% acrylamide gradient gel (Invitrogen, Carlsbad, CA, USA).

### 4.2. Western Blot

Western blots were performed with anti-apoE-HRP (1:1000) (Meridian Life Science, Inc., Memphis, TN, USA) or anti-acrolein lysine antibody (mAb5F6) (1:1000 dilution) [42] and HRP-conjugated anti-mouse IgG (1:5000 dilution) (Chemicon, Temecula, CA, USA), and detected using the enhanced chemiluminescence (ECL) detection kit (GE Healthcare, Uppsala, Sweden).

### 4.3. Circular Dichroism Spectroscopy

A Jasco 810 spectropolarimeter (Jasco Inc., Easton, MD, USA) was used to record CD data at 24 °C. Far-UV CD scans were recorded between 185 and 265 nm in 10 mM ammonium bicarbonate buffer, pH 7.4, containing 25 μM TCEP at a protein concentration of 0.2 mg/mL using a 0.1 cm path length circular cuvette.

The molar ellipticity ([θ]) in degrees × cm^2^ decimol^–1^ at 222 nm and the percent α-helix content were calculated as described earlier [59].

### 4.4. Mass Spectrometric Analysis

Unmodified and acro-apoE3 were subjected to mass spectrometric analysis as detailed under Supplemental Information. Briefly, about 10 µg protein samples were subjected to in-gel digestion and analyzed by nano LC-MS/MS with a Waters NanoAcquity HPLC system interfaced to a ThermoFisher Q Exactive (Waltham, MA, USA).

### 4.5. Preparation of rHDL

Unmodified or acro-apoE3 were reconstituted with POPC (at a phospholipid: protein mass ratio of 2.5:1) by the cholate dialysis method with slight modification [24]. The protein samples (4 mg) were pre-treated with 5× molar excess of tris(2-carboxyethyl)phosphine for 1 h at 24 °C to reduce intermolecular disulfide bonds prior to incubation with the lipid/detergent mixture. The samples were dialyzed for 48 h against PBS at 4 °C with three changes to remove cholate and promote formation of rHDL. Lipid-bound protein was separated from lipid-free protein and protein-free lipid vesicles by KBr density gradient ultracentrifugation at 230,000× *g* 10 °C for 5.5 h, fractionated and pooled as described earlier [24].

### 4.6. Non-Denaturing PAGE

To determine the size of apoE3/rHDL and acro-apoE3/rHDL, non-denaturing PAGE was carried out using 4–20% acrylamide gradient precast Tris-glycine gel (Invitrogen, Carlsbad, CA). Electrophoresis of ~ 10 μg sample was carried out along with high molecular weight protein standard markers (Amersham HMW Calibration Kit, G.E. Healthcare) in 10 mM Tris-glycine, pH 8.5 for 24 h at 132 V at 4 °C, and stained with Instant Blue (Expedeon, Cambridgeshire, UK).

### 4.7. Co-Immunoprecipitation

A co-immunoprecipitation (co-IP) assay was performed to determine the LDLr binding ability of apoE3/rHDL and acro-apoE3/rHDL. The details of the assay are provided under Supplemental Information.

### 4.8. DiI Labeling of rHDL

The lipid component of rHDL was labeled using the fluorescent 1,1′ – Dioctadecyl – 3,3,3′,3′ – tetramethylindocarbocyanine iodide (DiI, Invitrogen, OR, USA). About 50 µl of a stock solution of DiI (0.3 mg/mL) in DMSO (Alfa Aesar, HPLC grade, 99.9% Ward Hill, MA, USA) was incubated with apoE3/rHDL or acro-apoE3/rHDL (0.3 mg/mL) in PBS for 18 h at 37 °C in the dark. Unbound DiI was separated by density gradient ultracentrifugation as described above [60] and the sample filtered through 0.22 μm filter (Waters Millex HV units, SLHV R04 NL Millipore, Carrigtwohill, Co. Cork, Ireland) prior to application to cells.

### 4.9. Cellular Uptake of ApoE3/rHDL and Acro-apoE3/rHDL

Cellular uptake of apoE3/rHDL and acro-apoE3/rHDL was determined in bEnd.3 cells (5 × 10^5^ cells) (American Type Culture Collection, Manassas, VA, USA) in 6 well plates (Corning, Corning, NY, USA). Cells were grown in complete DMEM until they reached ~70% confluency, at which point the medium was replaced with pre-warmed 10% lipoprotein deficient serum (LPDS) in DMEM for an additional 24 h prior to addition of samples. Unlabeled or DiI-labeled apoE3/rHDL and acro-apoE3/rHDL (3 μg/mL in PBS) were incubated with bEnd.3 cells as such or in presence of 100× excess (300 μg/mL) LDL (MilliporeSigma) or ox-LDL for 2 h at 37 °C. Ox-LDL was prepared essentially as described previously [61] with slight modifications by incubating 50 µg/mL LDL (dialyzed to remove EDTA) in PBS with 200 µM CuSO_4_ for 20 h at 37 °C. This was followed by addition of 1 mM EDTA and 1 mM butyl-hydroxytoluene to terminate the reaction and dialysis against PBS. In other cases, cells were pre-treated with the following antibodies for 1 h at 37 °C prior to the addition of DiI-labeled apoE3/rHDL or acro-apoE3/rHDL: anti-CD36 (monoclonal, 100 μg/mL, Novus Biologicals, Centennial, CO, USA), anti-LOX1 (monoclonal, 100 μg/mL, gifted by Dr. T. Sawamura, Shinshu University, Nagano, Japan) or anti-SRB1 (polyclonal, 100 μg/mL, Novus Biologicals, CO, USA).

To visualize cellular uptake, the cells were fixed and analyzed by direct fluorescence. For immunofluorescence the cells were permeabilized with 0.2% Triton X-100 for 5 min at 24 °C, incubated with primary apoE antibody mAb3H1 (1:3000) for 1 h at 37 °C, followed by goat anti-mouse Alexa-555 labeled secondary antibody (1:3000) (Thermo Fisher Scientific, Waltham, MA, USA) for 1 h at 37 °C. In all cases the cells were stained with DAPI (4′,6-Diamidino-2-phenylindole dihydrochloride, Research Organics Inc., Cleveland, OH, USA), and representative confocal images shown.

## Figures and Tables

**Table 1:** Validated sites and nature of modification in acro-apoE3 following trypsin and elastase digestion and LC-MS/MS analysis *.

Trypsin					Elastase				
Site	Mass Increase	^†^ Best ASCORE	Spectral Count Acro-ApoE3	Nature of Lys Modification	Site	Mass Increase	^†^ Best Ascore	Spectral Count Acro-ApoE3	Nature of Modification
K1	-	-	-	-	K1	76	1000	3	MP-Lys
K69	76	161.94	9	MP-Lys ^§^	K69	76	1000	12	MP-Lys
K69	56	100.09	3	Propanal adduct	K69	56	100.09	2	Propanal adduct
K69	38	1000	1	Aldimine	K69	-	-	-	-
K72	76	119.87	9	MP-Lys	K72	76	153.17	15	MP-Lys
K72	38	1000	1	Aldimine	K72	38	1000	2	Aldimine
K75	76	1000	6	MP-Lys	K75	76	1000	8	MP-Lys
K75	94	1000	1	FDP-Lys ^††^	K75	94	1000	1	FDP-Lys
K75	56	120.17	2	Propanal adduct	K75	-	-	-	-
K75	38	1000	1	Aldimine	K75	-	-	-	-
K95	76	1000	5	MP-Lys	K95	76	1000	4	MP-Lys
K95	94	1000	1	FDP-Lys	K95	94	1000	1	FDP-Lys
K143	-	-	-	-	-	-	-	-	-
K146	-	-	-	-	-	-	-	-	-
K157	76	1000	6	MP-Lys	K157	76	1000	9	MP-Lys
K233	76	1000	9	MP-Lys	K233	76	1000	11	MP-Lys
K233	56	1000	4	Propanal adduct	K233	56	1000	2	Propanal adduct
K233	38	1000	1	Aldimine	K233	38	1000	1	Aldimine
K242	76	1000	4	MP-Lys	K242	76	1000	4	MP-Lys
K242	94	1000	2	FDP-Lys	K242	94	1000	2	FDP-Lys
K262	76	1000	6	MP-Lys	K262	76	1000	8	MP-Lys
K282	76	1000	1	MP-Lys	K282	76	1000	3	MP-Lys

* Shown are those sites identified only in acro-apoE3 (i.e., spectral count = 0 for corresponding site in unmodified apoE3) or for which the spectral score was higher in acro-apoE3 than that for unmodified apoE3. ^†^ Ascore is a measure of the probability of correct localization of acrolein-modified site determined as defined under Supplemental Information. The localization probability is 1 for all the validated sites. ^§^ MP-Lys: *N^ε^*-(3-methylpyridinium)-lysine; ^††^ FDP-Lys: *N^ε^*-(3-formyl-3,4-dehydropiperidino)lysine.

## Supplementary Materials

Supplementary materials can be found at https://www.mdpi.com/1422-0067/20/18/4582/s1.
