# Supplementary material for: Cellular Uptake and Clearance of Oxidatively-modified Apolipoprotein E3 by Cerebral Cortex Endothelial Cells
Source: Int J Mol Sci. 2019 Sep 17;20(18):4582. doi: 10.3390/ijms20184582 (PMC6769588; doi:10.3390/ijms20184582)
Supplement: Supplementary file 1 [file ijms-20-04582-s001.pdf]

Supplemental Information

Cellular Uptake and Clearance of Oxidatively-modified Apolipoprotein E3 by Cerebral Cortex Endothelial Cells

Siobanth Cruz, MS, and Vasanthy Narayanaswami, PhD

Department of Chemistry and Biochemistry

1250 Bellflower Blvd., California State University Long Beach, Long Beach, CA 90840, USA

Mass Spectrometric Analysis

About 10 μg of unmodified and acro-apoE3 were electrophoresed by SDS-PAGE using a 4-12% Bis-Tris NuPAGE gel in MOPS buffer (Invitrogen, Carlsbad, CA). The bands corresponding to 36 (and 72 kDa for acro-apoE3) were excised and subjected to in-gel digestion with trypsin or elastase, the former using a robot (ProGest, DigiLab); the gel pieces were washed with 25 mM ammonium bicarbonate and acetonitrile, reduced with 10 mM dithiothreitol at 60 ºC, alkylated with 50 mM iodoacetamide at 24 ºC, and digested with either trypsin or elastase (Promega Corporation, Madison, WI) for 4 h at 37 ºC at a 20:1 ratio (protein/protease, w/w). The digestion was quenched with formic acid and the supernatant analyzed directly (without further processing) by nano LC-MS/MS with a Waters NanoAcquity HPLC system interfaced to a ThermoFisher Q Exactive. The digested peptides were loaded on a trapping column and eluted over a 75 μm analytical column at 350 nl/min; both columns were packed with Luna C18 resin (Phenomenex). The mass spectrometer was operated in data-dependent mode, with the Orbitrap operating at. 70,000 FWHM and 17,500 FWHM for MS and MS/MS, respectively. The fifteen most abundant ions were selected for MS/MS.

The raw data were processed using Mascot (Matrix Science) or Byonic (Protein Metrics) (Cupertino, CA) MS/MS search engine and searched against the SwissProt database (human, appended with the target sequence bearing an N-terminal hexa-His tag and TEV protease specific site). The following variable modifications were specified: Acetyl (N-term), Deamidation (N/Q), Acrolein38 Delta:H(2)C(3) (K), Acrolein56 Delta:H(4)C(3)O(1) (K), Reduced Acrolein58 Delta:H(4)C(6) (K), Acrolein94 Delta:H(6)C(6)O(1) (K), Reduced Acrolein96 Delta:H(8)C(6)O(1) (K), with peptide mass tolerance of 10 ppm and fragment mass tolerance of 0.02Da. The Mascot DAT or Byonic MZID files were parsed into Scaffold (Proteome software) for validation, and filtered to create a non-redundant list per sample. Data were filtered at 1% protein and peptide false discovery rate and requiring at least two unique peptides per protein. Scaffold results were exported as mzidentML and imported into Scaffold PTM in order to assign site localization probabilities using Ascore (1). A minimum localization probability filter of 50% was applied.

Co-IP assay

The LDLr binding ability of apoE3/rHDL and acro-apoE3/rHDL was determined by a co-IP assay. A soluble construct bearing the essential LDLr ligand binding domains LA3-LA6 with a c-myc epitope (referred to as sLDLr) was used (2). ApoE3/rHDL or acro-apoE3/rHDL (10 μg) was incubated with 10 μg of sLDLr in the presence of 2 mM $CaCl_2$ in PBS for 1 h at 4 °C. This was followed by co-IP with anti c-*myc* antibody-linked agarose (MilliporeSigma, St. Louis, MO) to capture the receptor-bound complex and apoE3 detected by Western blot using HRP-conjugated polyclonal apoE antibody (1:5000 dilution) (Meridian Life Science, Inc., Memphis,

TN). To confirm the presence of sLDLr, a replica experiment was conducted wherein an anti c-myc antibody (9E10) (1:1000 dilution) (Abcam, Cambridge, UK) was utilized.

Unmodified ApoE3

Combined

417 exclusive unique peptides, 674 exclusive unique spectra, 1503 total spectra, 311/314 amino acids (99% coverage)

```
-15                      -1 1
HHHHHHENLY  FQGDPKVEQA  VETEPEPELR  QQTEWQSGQR  WELALGRFWD
YLRWVQTLSE  QVQEELLSSQ  VTQELRALMD  ETMKELKAYK  SELEEQLTPV
AEETRARLSK  ELQAAQARLG  ADMEDVCGRL  VQYRGEVQAM  LGQSTEELRV
RLASHLRKLR  KRLLRDADDL  QKRLAVYQAG  AREGAERGLS  AIRERLGPLV
EQGRVRAATV  GSLAGQPLQE  RAQAWGERLR  ARMEEMGSRT  RDRLDEVKEQ
VAEVRAKLEE  QAQQIRLQAE  AFQARLKSWF  EPLVEDMQRQ  WAGLVEKVQA
AVGTSAAPVP  SDNH
              299
```

Trypsin

55 exclusive unique peptides, 141 exclusive unique spectra, 806 total spectra, 302/314 amino acids (96% coverage)

```
-15                      -1 1
HHHHHHENLY  FQGDPKVEQA  VETEPEPELR  QQTEWQSGQR  WELALGRFWD
YLRWVQTLSE  QVQEELLSSQ  VTQELRALMD  ETMKELKAYK  SELEEQLTPV
AEETRARLSK  ELQAAQARLG  ADMEDVCGRL  VQYRGEVQAM  LGQSTEELRV
RLASHLRKLR  KRLLRDADDL  QKRLAVYQAG  AREGAERGLS  AIRERLGPLV
EQGRVRAATV  GSLAGQPLQE  RAQAWGERLR  ARMEEMGSRT  RDRLDEVKEQ
VAEVRAKLEE  QAQQIRLQAE  AFQARLKSWF  EPLVEDMQRQ  WAGLVEKVQA
AVGTSAAPVP  SDNH
              299
```

Elastase

380 exclusive unique peptides, 569 exclusive unique spectra, 697 total spectra, 306/314 amino acids (97% coverage)

```
-15                      -1 1
HHHHHHENLY  FQGDPKVEQA  VETEPEPELR  QQTEWQSGQR  WELALGRFWD
YLRWVQTLSE  QVQEELLSSQ  VTQELRALMD  ETMKELKAYK  SELEEQLTPV
AEETRARLSK  ELQAAQARLG  ADMEDVCGRL  VQYRGEVQAM  LGQSTEELRV
RLASHLRKLR  KRLLRDADDL  QKRLAVYQAG  AREGAERGLS  AIRERLGPLV
EQGRVRAATV  GSLAGQPLQE  RAQAWGERLR  ARMEEMGSRT  RDRLDEVKEQ
VAEVRAKLEE  QAQQIRLQAE  AFQARLKSWF  EPLVEDMQRQ  WAGLVEKVQA
AVGTSAAPVP  SDNH
              299
```

Acro-ApoE3

Combined

375 exclusive unique peptides, 668 exclusive unique spectra, 1508 total spectra, 311/314 amino acids (99% coverage)

```
-15                      -1 1
HHHHHHENLY  FQGDPKVEQA  VETEPEPELR  QQTEWQSGQR  WELALGRFWD
YLRWVQTLSE  QVQEELLSSQ  VTQELRALMD  ETMKELKAYK  SELEEQLTPV
AEETRARLSK  ELQAAQARLG  ADMEDVCGRL  VQYRGEVQAM  LGQSTEELRV
RLASHLRKLR  KRLLRDADDL  QKRLAVYQAG  AREGAERGLS  AIRERLGPLV
EQGRVRAATV  GSLAGQPLQE  RAQAWGERLR  ARMEEMGSRT  RDRLDEVKEQ
VAEVRAKLEE  QAQQIRLQAE  AFQARLKSWF  EPLVEDMQRQ  WAGLVEKVQA
AVGTSAAPVP  SDNH
              299
```

Trypsin

59 exclusive unique peptides, 179 exclusive unique spectra, 879 total spectra, 302/314 amino acids (96% coverage)

```
-15                      -1 1
HHHHHHENLY  FQGDPKVEQA  VETEPEPELR  QQTEWQSGQR  WELALGRFWD
YLRWVQTLSE  QVQEELLSSQ  VTQELRALMD  ETMKELKAYK  SELEEQLTPV
AEETRARLSK  ELQAAQARLG  ADMEDVCGRL  VQYRGEVQAM  LGQSTEELRV
RLASHLRKLR  KRLLRDADDL  QKRLAVYQAG  AREGAERGLS  AIRERLGPLV
EQGRVRAATV  GSLAGQPLQE  RAQAWGERLR  ARMEEMGSRT  RDRLDEVKEQ
VAEVRAKLEE  QAQQIRLQAE  AFQARLKSWF  EPLVEDMQRQ  WAGLVEKVQA
AVGTSAAPVP  SDNH
              299
```

Elastase

334 exclusive unique peptides, 523 exclusive unique spectra, 629 total spectra, 303/314 amino acids (96% coverage)

```
-15                      -1 1
HHHHHHENLY  FQGDPKVEQA  VETEPEPELR  QQTEWQSGQR  WELALGRFWD
YLRWVQTLSE  QVQEELLSSQ  VTQELRALMD  ETMKELKAYK  SELEEQLTPV
AEETRARLSK  ELQAAQARLG  ADMEDVCGRL  VQYRGEVQAM  LGQSTEELRV
RLASHLRKLR  KRLLRDADDL  QKRLAVYQAG  AREGAERGLS  AIRERLGPLV
EQGRVRAATV  GSLAGQPLQE  RAQAWGERLR  ARMEEMGSRT  RDRLDEVKEQ
VAEVRAKLEE  QAQQIRLQAE  AFQARLKSWF  EPLVEDMQRQ  WAGLVEKVQA
AVGTSAAPVP  SDNH
              299
```

Figure S1. Sequence coverage map of unmodified and acro-apoE3. The amino acid sequence of apoE3 (1-299) is shown; the His-tag and TEV protease cleavage sites at the N-terminal end of

the protein are numbered -15 to -1. Peptide segments obtained by trypsin or elastase treatment are shown for mass spectral analysis. Also shown are the sites covered by both treatments under ‘Combined’. Green highlights indicate PTM sites identified by mass spectrometry, including deamidation and sulfoxide derivatives.

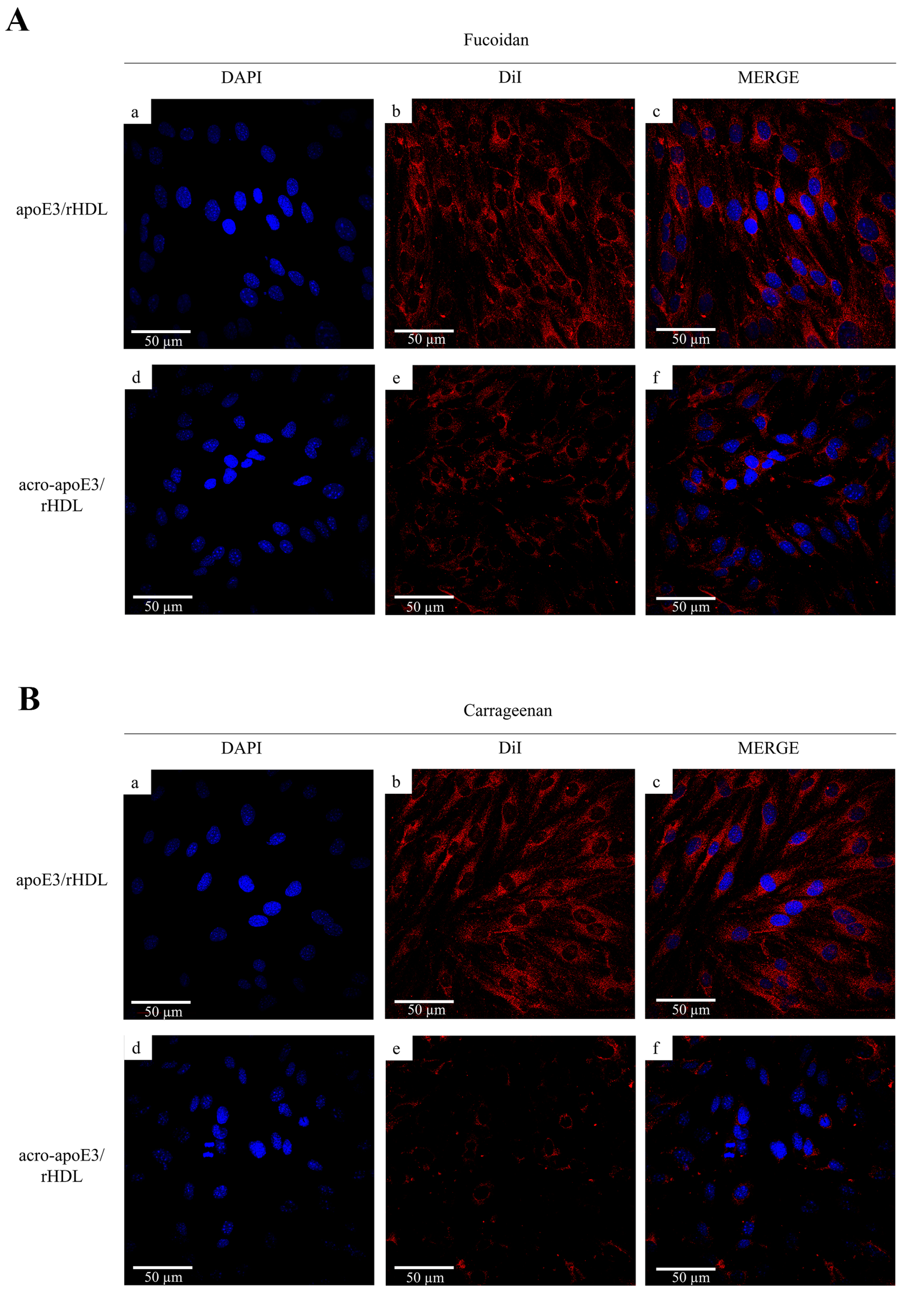

Figure S2. A. Uptake of apoE3/rHDL and acro-apoE3/rHDL in the presence of fucoidan or carrageenan in bEnd.3 cells. Uptake experiments were carried out for 2 h at 37 °C with 3 µg/ml apoE3/rHDL (a-c) or acro-apoE3/rHDL (d-f) in the presence of 0.25 mg/ml fucoidan (A) or 0.25 mg/ml carrageenan (B). The panels show fluorescence of DAPI (a and d), DiI (b and e), and Merge (c and f). Representative confocal images are shown.

References

1. Beausoleil SA, Villén J, Gerber SA, Rush J, Gygi SP. A probability-based approach for high-throughput protein phosphorylation analysis and site localization. *Nat. Biotechnol.* 2006;24: 1285–1292

2. Fisher C, Abdul-Aziz D, Blacklow, SC. A two-module region of the low-density lipoprotein receptor sufficient for formation of complexes with apolipoprotein E ligands, *Biochemistry.* 2004;*43*:1037-1044
